# Diversity of Zoanthids (Anthozoa: Hexacorallia) on Hawaiian Seamounts: Description of the Hawaiian Gold Coral and Additional Zoanthids

**DOI:** 10.1371/journal.pone.0052607

**Published:** 2013-01-09

**Authors:** Frederic Sinniger, Oscar V. Ocaña, Amy R. Baco

**Affiliations:** 1 Submarine Resources Project, Japan Agency for Marine-Earth Science and Technology, Yokosuka, Kanagawa, Japan; 2 Global Oceanographic Data Center, Japan Agency for Marine-Earth Science and Technology, Nago, Okinawa, Japan; 3 Marine Biodiversity Program, Institute of Biogeoscience, Japan Agency for Marine-Earth Science and Technology, Yokosuka, Kanagawa, Japan; 4 Tropical Biosphere Research Center, University of the Ryukyus, Motobu, Okinawa, Japan; 5 Departamento de Biologia Marina, Fundacion Museo del Mar, Ceuta, North Africa, Spain; 6 Department of Oceanography, Florida State University, Tallahassee, Florida, United States of America; Heriot-Watt University, United Kingdom

## Abstract

The Hawaiian gold coral has a history of exploitation from the deep slopes and seamounts of the Hawaiian Islands as one of the precious corals commercialised in the jewellery industry. Due to its peculiar characteristic of building a scleroproteic skeleton, this zoanthid has been referred as *Gerardia* sp. (a junior synonym of *Savalia* Nardo, 1844) but never formally described or examined by taxonomists despite its commercial interest. While collection of Hawaiian gold coral is now regulated, globally seamounts habitats are increasingly threatened by a variety of anthropogenic impacts. However, impact assessment studies and conservation measures cannot be taken without consistent knowledge of the biodiversity of such environments. Recently, multiple samples of octocoral-associated zoanthids were collected from the deep slopes of the islands and seamounts of the Hawaiian Archipelago. The molecular and morphological examination of these zoanthids revealed the presence of at least five different species including the gold coral. Among these only the gold coral appeared to create its own skeleton, two other species are simply using the octocoral as substrate, and the situation is not clear for the final two species. Phylogenetically, all these species appear related to zoanthids of the genus *Savalia* as well as to the octocoral-associated zoanthid *Corallizoanthus tsukaharai*, suggesting a common ancestor to all octocoral-associated zoanthids. The diversity of zoanthids described or observed during this study is comparable to levels of diversity found in shallow water tropical coral reefs. Such unexpected species diversity is symptomatic of the lack of biological exploration and taxonomic studies of the diversity of seamount hexacorals.

## Introduction

Deep-sea corals, particularly on seamounts, have received significant attention over the last decade [1]. These corals have been shown to play a role as ecosystem biobuilders, acting as habitats for diverse invertebrate and fish communities [2]. In these studies, significant attention is given to scleractinians and more recently to octocorals. However, as in shallow water, the hexacorallian order Zoantharia (also sometimes called Zoanthidea), may also make up a considerable component of some deep-sea coral communities [3], [4], [5] but have received little attention.

The order Zoantharia is characterised by two rows of tentacles and, most often, a colonial mode of life. This order is commonly referred as “zoanthids” in English despite the possible confusion with the family Zoanthidae, a group within this order, restricted to shallow tropical and subtropical environments. In this paper, we follow the most used standards in past and present specialised taxonomic literature, using Zoantharia as the scientific order name and “zoanthids” as the common name for the order. Zoanthids can be found in most marine environments although very little is known of their ecological role in the ecosystem. Deep-sea zoanthids remain largely unknown with the exception of a few species regularly dredged or trawled on deep-sea soft sediments. Most of these zoanthids belong to the genus *Epizoanthus* Gray, 1867, in the family Epizoanthidae Delage and Hérouard, 1901, with a few species associated with pagurids [6] and some associated with hexactinellid sponge stalks. *Isozoanthus* Carlgren in Chun, 1903 within the family Parazoanthidae Delage and Hérouard, 1901 is the second most speciose genus of deep-sea zoanthid species, (however recent taxonomic work casts doubt on the status of this genus [7]). Another group of deep-sea zoanthids was recently described from over 3000 m depth in a cold seep environment, the family Abyssoanthidae Reimer and Fujiwara in Reimer et al., 2007. Recently specimens belonging to this group have been found in other bathyal and abyssal environments around the world [8], [9]. Between 100 m and 2000 m depth, numerous epizoic zoanthids, usually belonging to the family Parazoanthidae, can be found. However, most of them remain undescribed despite some species such as the Hawaiian gold coral (often referred as *Gerardia*) having been known for decades. Table 1 reviews the currently recognised zoanthid genera and families with an indication of the genera found in the deep-sea.

**Table 1:** Classical taxonomic organisation within the order Zoantharia.

Sub-Order	Family	Genus
Brachycnemina	Neozoanthidae	*Neozoanthus*
	Sphenopidae	*Palythoa, Sphenopus*
	Zoanthidae	*Acrozoanthus, Isaurus, Zoanthus*
Macrocnemina	Epizoanthidae	*Epizoanthus* *, *Paleozoanthus, (Thoracactis)* ** a
	Hydrozoanthidae	*Hydrozoanthus, Terrazoanthus*
	Microzoanthidae	*Microzoanthus*
	Parazoanthidae	*Isozoanthus* *, *Corallizoanthus* **, *Antipathozoanthus, Mesozoanthus* *, *Parazoanthus* *, *Savalia* *
Incertae Sedis	Abyssoanthidae	*Abyssoanthus* **

a Only one sample is known from that genus initially described as an actiniarian and it was not conserved well enough for genus, family or even suborder identification. A recent confusion appeared with the use of *Thoracactus* by Walsh in 1967, however here we consider the original denomination correct.

* genera found both in shallow and deep (subphotic) environments.

** genera exclusively found in deep (subphotic) environments.

### Hawaiian gold coral

**Figure 1:**
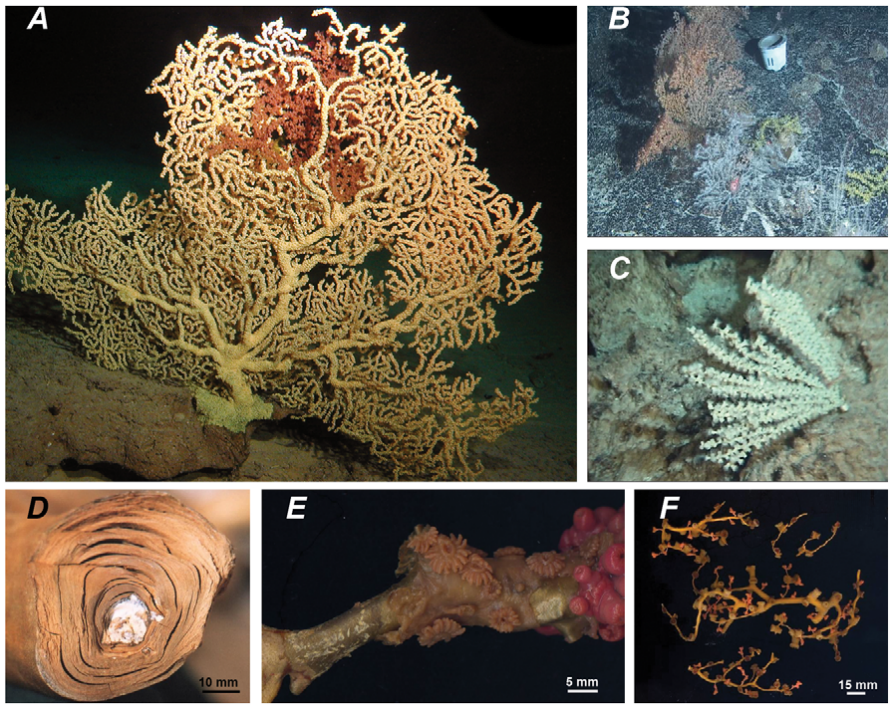
Gold coral and related zoanthids from Hawaii. A) *Kulamanamana haumeaae* gen. n. sp. n. in situ, B) *Zibrowius ammophilus* gen. n. sp. n. in situ, C) *Hurlizoanthus parrishi* gen. n. sp. n. in situ, D) axis of *Ku. haumeaae* with the calcified skeleton of host bamboo coral visible in the center, E) *Kauluzoanthus kerbyi* gen. n. sp. n. fixed sample, polyps of *Ku. haumeaae* can be seen on the right coloured in pink after reacting with formaldehyde, F) *Bullagummizoanthus emilyacadiaarum* gen. n. sp. n. fixed sample.

The Hawaiian gold coral (fig. 1A) is a zoanthid found in the Hawaiian Archipelago at depths ranging from 343 m to 575 m. This species was discovered in 1970 following investigations of *Corallium secundum* Dana, 1846 [10]. It is one of the few zoanthids able to secrete a scleroproteic skeleton (fig. 1D). Its name “gold coral” originates in the golden colour of this secretion. This peculiarity has been used in jewellery and led to the commercial exploitation of the Hawaiian gold coral from the 1970's until 2001 with an interruption between 1979 and 1999 [11]. The gold coral is not currently exploited in Hawaii due to a restriction on the fishery which requires selective harvest making collection of this species cost prohibitive. Besides its role in the fishery, the Hawaiian gold coral is one of the largest and numerically dominant benthic macro-invertebrates in its depth range on hard substrate habitats of the Hawaiian Archipelago. Thus, the Hawaiian gold coral plays an important ecological role in Hawaiian seamount benthic assemblage [3], [5].

The Hawaiian gold coral may also be one of the longest-lived species on earth. The skeleton and commercial interest in this species led to multiple studies attempting to date the age of the colonies. Earlier ageing attempts on the gold coral focused on ring counts [10], [11] and led to a maximal estimated age of 70 years and a radial growth rate (increase in branch diameter) of 1 mm/year. Recent studies on the red coral *Corallium rubrum* (Linnaeus, 1758) [12] suggested that the rings traditionally observed based on the density of the skeleton may not reflect yearly cycles but much longer time intervals. This is supported for the Hawaiian gold coral by recent studies using radiometric data which suggest colonies of Hawaiian gold coral are as old as 2740 year with a radial growth rate of 15 to 45 µm/year [13], [14], [15]. In the Atlantic a similar zoanthid referred to as “*Gerardia*” is found and amino acid racemization experiments [16] indicated a maximal age of 250 years while radiocarbon dating estimate the life span of a colony to 1800 years [17].

### Hawaiian gold coral nomenclature

Despite its ecological significance and long history of exploitation, the Hawaiian gold coral has never been subject to taxonomic studies or a formal species description. As a result of this, the nomenclature concerning the Hawaiian gold coral has been relatively confused. This species was first mentioned as *Parazoanthus* sp. [10] before being referred to as *Gerardia* Lacaze-Duthiers, 1864 starting in 1976 in ageing studies [18], due to its secretion of a scleroproteic skeleton. However, the genus *Gerardia* had been recognised as a younger synonym from the genus *Savalia* Nardo 1844 already [19]. More recently this synonymy was mentioned in several publications [20]–[22].

Symptomatic of the order, a suite of other zoanthids, besides the Hawaiian gold coral, have been observed and collected in Hawaii, but far less is known of their biology and ecology and they have not been described taxonomically.

### Zoanthid taxonomy

In 1913 Carglren, who was probably the most active researcher on this order, said “Among the Anthozoa (…) there is hardly a group which is so uniform in its morphological characteristics as the Zoantharia …” [23]. Nearly a hundred years later, despite numerous taxonomical investigations of the morphological characteristics of this order, Carlgren's statement is still accurate. The sphincter position has been traditionally used to identify zoanthid genera, although Lwowsky [24] illustrated the risks of misidentification using this character. This is exemplified in the Parazoanthidae, in which recent taxonomic work casts doubts on the significance of the sphincter muscle position (the main distinguishing feature of *Isozoanthus*) as a valid character [7] and recent studies based on morphology assigned various unrelated zoanthids to the genus *Isozoanthus* [25]–[28], none of which matched with the ecological characteristics of previously described *Isozoanthus* species. Similarly, Swain [29] showed clearly that the sphincter position did not allow proper identification at the genus level and does not represent the evolutionary history of this group.

At the species level, nematocysts appear to be the most promising morphological character for use within the order [28], [30], [31]. However, Ryland et al. [32] suggests using very large sample size to obtain reliable information, although such size are too large for most descriptions of new species (which are often represented by only a few polyps). Additionally, until now no studies have been made on the intraspecific and intra-individual variation of nematocysts sizes and assemblages in zoanthids through their life cycle and through changes in biological activity. For these reasons, nematocysts as a diagnostic character are to be interpreted with caution until more research is being made both on intra- and interspecific cnidome variations.

Recently, DNA information has shed light on the phylogenetic relationships among zoanthids and has also helped revise the taxonomy of several groups of zoanthids as well as describe new taxa [7], [8], [21], [33]–[35]. However, the sole use of DNA to identify zoanthid species might not be sufficient to distinguish closely related species [36] and it is therefore necessary to integrate not only DNA and morphology but also ecological parameters to identify specimens.

### Epizoism

Within the suborder Macrocnemina (comprising all the specimens examined here) most species are epizoic. Recent molecular phylogenetic studies suggest a possible relation between the group of organisms used as substrate and the evolution of the suborder [7], [21]. This hypothesis concerns essentially the family Parazoanthidae. Species within the other commonly epizoic family, Epizoanthidae, are often associated with gastropod shells (regularly inhabited by pagurid crustaceans) or tube worms, and do not appear to form monophyletic groups according to the type of substrate used [37]. Parazoanthid species on the other hand, do appear to form monophyletic groups correlated to the organisms colonized (such as sponges, antipatharians or octocorals) and a recent revision of the family used this character to help redefine genera [7]. Most species associated with hydrozoans were recently separated from Parazoanthidae to be placed in their own family Hydrozoanthidae based on the phylogenetic information [7]. However, as mentioned in Ocana et al. [22] other zoanthids can occasionally be found on hydrozoans. Therefore, and especially for octocoral-associated zoanthids, the low number of specimens available for studies led to prudence in the interpretation of the substrate specificity hypothesis. Until now only four species associated with octocorals have been described, all in the family Parazoanthidae; *Savalia savaglia* [38] from the Mediterranean Sea and East Atlantic, *S. lucifica* [39] from California (originally described as *Parazoanthus lucificum*), *Corallizoanthus tsukaharai* [35] from Japan and *Isozoanthus primnoidus* [28] from the Azores. The Hawaiian zoanthids and a gold-coral related species from the Bahamas represent additional known, yet undescribed, zoanthids presumably associated with octocorals.

The purpose of this study is to re-examine specimens of the Hawaiian gold coral and four other species of arborescent zoanthids from the Hawaiian Archipelago to evaluate the biodiversity of these ecologically important zoanthids and to provide taxonomic placement of these species in consideration of their evolutionary history. This aim was achieved and results in the description of several new zoanthid genera, a significant advance in the understanding of zoanthid biodiversity considering that until this study only 18 genera were recognised.

## Materials and Methods

### Sampling

Samples were collected using Hawaiian Undersea Research Laboratory (HURL) submersibles *Pisces IV and V*, during cruises in 1998–2004. All necessary permits were obtained for the described field studies. Zoanthids collected in the Northwestern Hawaiian Islands were collected when it was the NWHI Coral Reef Ecosystem Reserve under permit # NWHICRER-2003-003 and -004. Collections were made prior to establishment of the Papahanaumokuakea Monument. CITES does not apply as zoanthids are not listed in CITES. Where possible, but consistently from 2003 on, each colony was filmed *in situ* before being sampled. Samples were fixed with 8–10% formaldehyde and later stored in 70% alcohol in the Baco-Taylor lab. A subsample of each specimen was also stored at -80 or in 95% ethanol for genetics. Types specimens were separated into several schizotypes depending on the fixation methods and to facilitate access to the type material to researchers worldwide. All the type material is deposited at the MNHG (Natural History Museum of Geneva, Switzerland) and USNM (National Museum of Natural History; Smithsonian Institution; Washington, DC, USA).

### Morphological and cytological examinations

General morphology and anatomy were studied by means of a stereo dissecting microscope. The anatomical and micro anatomical details were studied using staining *in toto*. Nematocysts were examined with a light microscope equipped with a Nomarski differential interference contrast optic system. A minimum of ten polyps for each species and all the colonies available were examined. The classification and terminology of nematocysts follows that of Schmidt [40], as adapted by den Hartog [41] and den Hartog *et al*. [42], in table 2 we also include the terminology used by Ryland and Lancaster [43]. The surveys of the cnidome are summarised in table 2 in which the ranges of length and width of nematocysts are included.

**Table 2:** Distribution, type, relative abundances and size ranges of cnidae in the new zoanthids species described here.

Tissue	Nematocyst types	*Ku*. *haumeaae* gen. n. sp. n.	*Z*. *ammophilus* gen. n. sp. n.	*H*. *parrishii* gen. n. sp. n.	*Ka*. *kerbyii* gen. n. sp. n.	*B*. *emilyacadiaarum* gen. n. sp. n.
Tentacles	Spirocysts	vc; 15–30×3–4 (A)	vc; 16–30×2.5–4 (A)	vc; 15–25×2.5–4 (A)	vc; 20–35×3–4 (A)	vc; 20–35×3–5 (A)
	Spirulae	rc-c; 20–27×3.5–4.5 (B)	c; 17–21×4–5 (B)	rc; 18–21×3.5–4 (B)	c; 16–23×2.5–3 (B)	vc; 20–30×3–5 (B)
	Special Spirulae				rc; 12–16×3–4 (C)	
	Penicilli			r; 14–20×5–6		
	Penicilli A	u; 25–35×6–7 (C)				
	Penicilli E	c; 18–25×8–11 (D)			u; 30–35×15–21 (D)	rc; 27–35×11–15 (C)
	Homotrichs 1*	c-vc; 11–15×4–5 (E)	rc; 9–12×3–4 (C)	u; 10–12×3–4 (C)		rc; 9–13×3.5–4 (D)
	Homotrichs 2*					rc; 8–12×2.5–3 (E)
Pharynx	Spirulae	rc-c; 20–25×3–5 (F)	u; 16–18×4 (D)	rc-uc; 16–30×3.5–7 (D)	vc; 18–24×2.5–4 (F)	c; 20–24×4–4.5 (F)
	Penicilli A	rc-u; 20–25×5–7 (G)	rc; 18–21×5–7 (E)	r; 17–22×4–6 (E)		u-rc; 19–21×5–6 (G)
	Penicilli E	c-vc; 18–23×8–10 (H)				rc; 24–32×11–15 (H)
	Penicilli E Special		r; 32×5 (F)			
	Homotrich 1*	u-rc; 12–15×3–4 (I)	rc-c; 9–11×3–4 (G)	r; 13–18×3–5 (F)		c-vc; 12–14×4–5 (I)
	Homotrich 2*			u; 10–12×1.5–4 (G)		c-vc; 11–14×3–3.5 (J)
Filaments	Spirulae			r; 20–23×4 (H)		u-rc; 19–25×3.5–4 (P)
	Special Spirulae		u; 12–13×6–7 (H)			u; 20–22×13–15 (Q)
	Penicilli			r; 15–17×8–10 (I)		
	Penicilli A	c; 20–26×6–7 (J)	c; 16–20×5–6 (I)	rc-c; 18–25×4.5–6 (J)	c; 16–25×5–6 (G)	c-vc; 17–24×5–7 (R)
	Penicilli E	c-vc; 20–23×8.5–10 (K)	rc; 27–30×11–16 (J)			u-rc; 27–35×12–16 (S)
	Penicilli E Special					c; 30–50×5–7 (T)
	Homotrichs 1*	uc-rc; 10–15×3–5 (L)		u-rc; 10–15×3–4 (K)	rc; 12–19×2–3 (H)	c-vc; 11–14×4–6
	Homotrichs 2*					c-vc; 10–15×4–5
	Homotrichs 3*					r; 15×3
Body wall	Spirulae	r; 22–27×3.5–4 (M)				u-rc; 20–26×3.5–4 (K)
	Special Spirulae					c; 18–25×13–19 (L)
	Penicilli A	u-rc; 29–40×6–8 (N)				
	Penicilli E	c-vc; 21–25×8–10 (O)	u; 32–40×18–25 (K)	rc-c; 43–55×18–23 (L)	uc; 35–38×15–20 (I)	vc; 21–30×11–16 (M)
	Homotrichs 1*	rc; 12–16×3.5–4 (P)	u; 8–9×2.5–3 (L)	c; 10–15×3–4 (M)	c; 10–12×1.5–2 (J)	rc-c; 10–12×3.5–4.5 (N)
	Homotrichs 2*					rc-c; 11–15×4–5 (O)

Abbreviations: r: rare, u: uncommon, rc: relatively common, c: common, vc: very common. Capital letters refer to figure 3. Number ranges indicate cnidae dimensions (length x width) in µm.

* Several types of small homotrichs are distinguished; however they might be extremes of a wide morphological variation range of a single type.

Cnidae nomenclature differs according to different authors. Here is a list of correspondant terms: Spirulae ( = b-mastigophore; Basitrichs), Penicilli ( = P-mastigophore), Penicilli A ( = p-mastigophore A), Penicilli E ( = Holotrichs; Homotrichs; p-mastigophores E), Penicilli E Special (p-mastigophore E special; Homotrichs, Holotrichs), Homotrichs (Holotrichs).

### DNA processing and analyses

DNA was extracted following the methods described in Sinniger et al. [7]. COI, mt 16S rDNA and nuclear 18S rDNA were then amplified using primers LCOant, HCOantr, 16Sant0a [7], 16SbmoH [21], 18SA, 18SB [44], 18SC, 18SL, 18SO, 18SY [45] and standard Taq polymerase (several manufacturers such as Qiagen, Invitrogen, Roche and Takara were used without any observed effect on the results). Sequencing was either sent to a commercial sequencing company or carried out using a BigDye Terminator Cycle Sequencing Ready Reaction Kit following the manufacturer instructions (Applied Biosystems). Sequences were then run on an ABI-3100 Avant automatic sequencer. Newly obtained sequences were deposited on GenBank (KC218386-KC218440). GenBank accession numbers detailed for each specimen are reported in Table S1. Sequences for both strands were manually assembled and chromatograms were checked for quality. Resulting sequences were manually aligned using BioEdit ver. 5.0.9 [46]. After individual alignment for each marker, a multigene alignment including the three markers was created. This alignment was partitioned and analysed with the maximum likelihood (ML) method using Treefinder [47]. Bayesian analyses were performed using MrBayes ver. 3.0 [48] Analyses were performed with GTR nucleotide substitution matrix, a gamma 1 invariant model with eight categories, and for each marker α-parameter and frequencies of nucleotides were estimated independently. This method was chosen as it allows the most complete estimation of parameters. Species belonging to the macrocnemic family Epizoanthidae and family Hydrozoanthidae were used as outgroups. Sequences of *Abyssoanthus* were not included in the analyses due to their short length and high divergence. Only zoanthid outgroups were used in order to keep a maximum of informative sites in the alignments (the insertion-deletion pattern of other hexacorallian orders were too divergent to allow reliable alignment construction in mt 16S rDNA).

### Nomenclatural acts

The electronic edition of this article conforms to the requirements of the amended International Code of Zoological Nomenclature, and hence the new names contained herein are available under that Code from the electronic edition of this article. This published work and the nomenclatural acts it contains have been registered in ZooBank, the online registration system for the ICZN. The ZooBank LSIDs (Life Science Identifiers) can be resolved and the associated information viewed through any standard web browser by appending the LSID to the prefix “http://zoobank.org/”. The LSID for this publication is: urn:lsid:zoobank.org:pub: 369F0BC5-9CDD-496E-8C21-2BAA9E996531. The electronic edition of this work was published in a journal with an ISSN, and has been archived and is available from the following digital repositories: PubMed Central, LOCKSS.

## Results

### Taxonomic section

Abbreviation used: 16S = mitochondrial large ribosomal subunit; ARB = Amy Baco; BPBM = Bernice Pauahi Bishop Museum; MMC = Museo del Mar, Ceuta (Spain); MNHG = Natural History Museum of Geneva (Switzerland); NIWA = National Institute of Water and Atmospheric Research (Wellington, New Zealand); NSMT = National Museum of Nature and Science (Tokyo, Japan); USNM = Smithsonian Institution (Washington DC, USA); RMNH = Naturalis (Leiden, The Netherlands).

Suborder Macrocnemina Haddon & Shackleton, 1891.

Zoanthids characterised by a complete fifth pair of mesenteries.

Family Parazoanthidae Delage & Hérouard, 1901

Macrocnemic zoanthids with an endodermal sphincter (according to the original description).

*Kulamanamana* gen. n.

urn:lsid:zoobank.org:act:F1F3FE5D-3482-4658-A84B-589D2C4E225E

Type species: *Kulamanamana haumeaae* sp. n.

Etymology: the feminine name of this genus is derived from the Hawaiian terms Kula ( = gold) and manamana ( = branch) referring to the particular skeleton of the type species of this genus.

**Figure 2:**
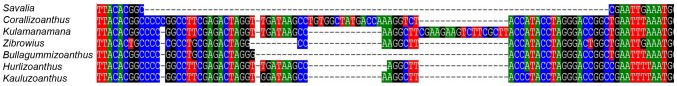
Genetic signatures of *Savalia* and related zoanthid genera. This figure shows the V5 region sensu Sinniger et al. [21] with characteristic insertion/deletion pattern specific to each genus. This region is located in the second half of the mt16S rDNA gene. Sequences are represented from 5′ to 3′ and the sequences used are AY995925, EU035623, KC218431, KC218438, KC218434, KC218433, KC218435.

Diagnosis: Macrocnemic genus associated with octocorals secreting a golden to dark brown scleroproteic skeleton. Absence of mineral incrustations in the ectoderm, well developed coenenchyme completely covering the host,. Characteristic insertion/deletion pattern in the 16S V5 region sensu Sinniger et al. [21] (fig. 2).

*Kulamanamana haumeaae* sp. n. (Hawaiian gold coral)

**Figure 3:**
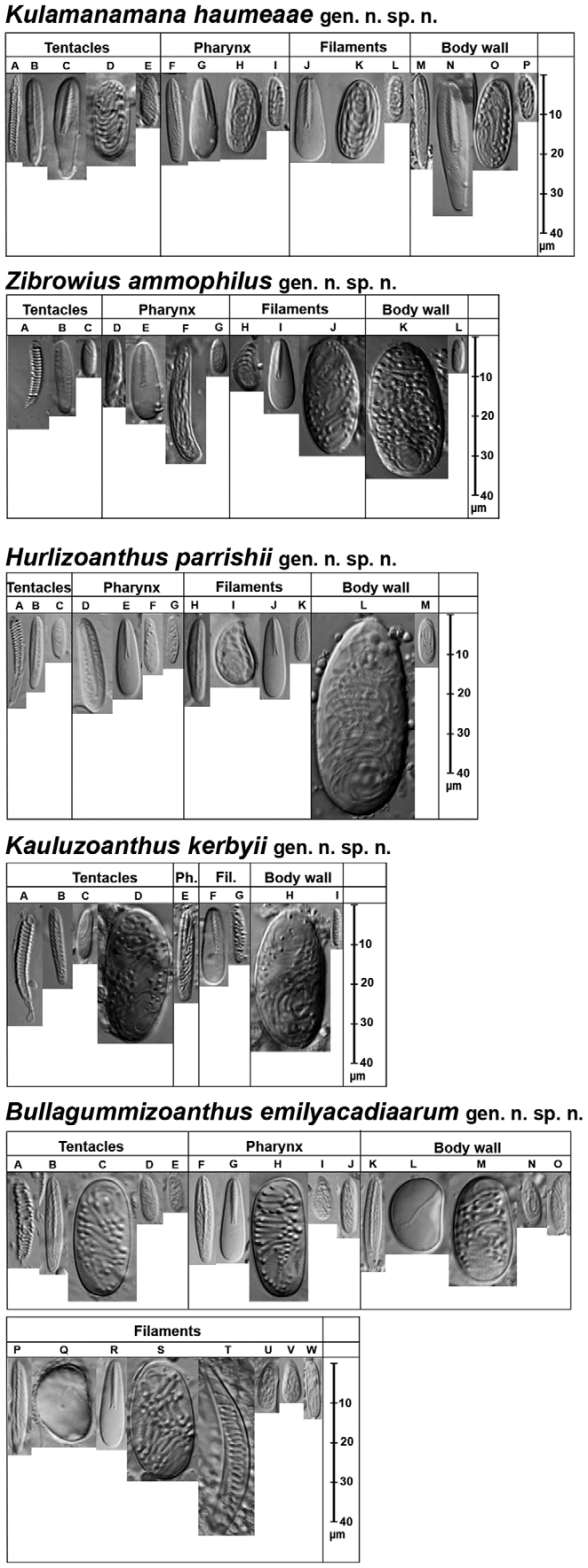
Cnidae of the different species. Cnidae in the different tissues of the zoanthids described here. Letters correspond to the cnidae listed in table 2.

Figs. 1 A, D, E, 3 (top)

urn:lsid:zoobank.org:act:DE4B202A-3564-4111-8885-81B5AC0DBCE7

Synonyms: *Parazoanthus* sp. [10], *Gerardia* sp. [1], [3]–[5], [11], [13]–[15], [18], [49]–[58], *Savalia* sp. [59].

Etymology: this species name is dedicated to Haumea, Hawaiian goddess of fertility.

Material examined:

Holotype: P5 593, Keahole Point (19°48.203′-19°47.943′ N, 156°08.047′-156°07.478′ W), Hawaii, 15.10.2004, 396.5 m, coll. ARB, more than 200 polyps on fragments of different sizes, presence of some polyps of the parasitic zoanthid *Kauluzoanthus kerbyi*, USNM 1190187 (formalin-fixed), fragment of 20–25 small polyps, USNM 1190188 (ethanol-fixed).

Paratypes: P5-582, Makapuu, Hawaii, 02.10.2004, 395 m, two fragments of 30–35 and 20 polyps of small size, BPBM-D2250 (ethanol-fixed); P5-582, Makapuu, Hawaii, 02.10.2004, 401 m, three fragments of 18–20, 15 and 25–30 polyps medium to large size, MNHG-INVE-82279 (ethanol-fixed); P5-583, Makapuu, Hawaii, 03.10.2004, 427 m, three fragments of 24, 30–35 and 10–15 polyps of different sizes, NIWA-84101 (ethanol-fixed); P5-583, Makapuu, Hawaii, 03.10.2004, 432 m, two fragments of 90–95 and 30–35 polyps of different sizes, MMC-T3 (ethanol-fixed); P5-583, Makapuu, Hawaii, 03.10.2004, 406 m, two fragments from the same colony of 45–50 and 60–65 polyps of different sizes, MMC-T4 (formalin-fixed); P5-585, Lanikai, Hawaii, 05. 10. 2004, 400 m, fragment of 10 small polyps and also a fragment of 85–90 polyps of small size, USNM 1190189 (formalin-fixed); P5-586, Lanikai, Hawaii, 05.10.2004, 410 m, three fragments of, 10–15, 10–15 and 30–35 polyps of different sizes, NIWA-84102 (ethanol-fixed); P5-588, Cross Seamount, Hawaii, 09.10.2004, 394 m, three fragments of 15–20 and 20–25 polyps of medium to big sizes, NSMT-Co 1547 (ethanol-fixed), two fragments of 8–10 and 20–25 polyps of medium size, MNHG-INVE-82280 (ethanol-fixed); P5-589, Cross Seamount, Hawaii, 09.10.2004, 388 m, two fragments from the same colony of 80–90 polyps of different sizes and 25–30 polyps of small size, BPBM-D2251 (ethanol-fixed); GP5 P5-523, Bank 8, Hawaii, 06.10.2003, 526 m, USNM 1190190 (ethanol-fixed); GP6 P5-522, Bank 8, Hawaii, 05.10.2003, two fragments of 75–80 and 35–40 polyps of different sizes, 544 m, NSMT-Co 1548 (ethanol-fixed); P4-19, Keahole point, Hawaii, 22.11.2000, 385 m, fragment of 55 polyps of equal size, RMNH Coel. 40076 (ethanol-fixed). All samples collected by ARB.

Sequences: 18S: KC218399, KC218400, KC218401; 16S: KC218431, KC218432; COI: KC218386, KC218387, KC218388.

Diagnosis: Golden axis, tissue colour ranging from pale yellow to medium orange, secretion of excessive mucus when collected and absence of mineral incrustations are distinctive characters of this species, In terms of cnidome, the main diagnostic characters appear to be the presence of enlarged penicilli A (p-mastogophores A) in tentacles and body wall and penicilli E in all the tissues.

External morphology: In preserved specimens, size of contracted and semi-contracted polyps from 1–8 mm length and 2–7 mm diameter. 28–31 pointed tentacles (usually 28). In expanded living polyps, length of the tentacles equivalent to up to three quarters of the diameter of the oral disc. 13–16 bractea (corresponding to capitular ridges on closed polyps) on the edge of the oral disc. Apex of closed polyps flat (but might appear rounded in small or extremely contracted polyps). Column cylindrical, with no incrusted particles. Well developed coenenchyme completely covering the host. Polyp density variable within the colony with coenenchyme barely visible in the most dense regions (usually the smallest branches) and polyps separated by over 1 cm in the less dense area (such as the main stem). In situ characterised by bioluminescence when disturbed. Size of the fan-shaped colonies up to over 2 m diameter and over 1 m height. Golden scleroproteic skeleton, distinct from the black skeleton found in the genus *Savalia* Nardo 1844 ( = *Gerardia* L.-D. 1864). In most major parts of the skeleton, host axis can be observed in the centre. Anastomosed branches are frequently observed and the complex branching pattern and larger size of the colonies is clearly distinct from that of the isidids or other octocorals found in the area, suggesting the ability of this zoanthid to build its own branches (fig. 1a). As observed in other epizoic genera (*Savalia* and *Antipathozoanthus* particularly) the colony can spread on other substrata at the base of the original host.

Colour: In vivo, polyps, tentacles and coenenchyme bright yellow to dark orange. Upon formalin fixation tissues turns red as observed in several other zoanthid species. Bioluminescence has been observed in situ.

Microanatomy: 25 to 30 mesenteries following macrocnemic arrangement. Most mesenteries fertile, including incomplete mesenteries. Retractor muscle absent, parietobasilar muscle developed and forming visible pennons. Single siphonoglyph prominent. Sphincter endodermal (0.1–0.3 mm), concentrated in the upper part of the column, forming sinus and processes.

Cnidome: spirocysts, spirulae (b-mastigophores, basitrichs), small homotrichs (holotrichs); penicilli A (p-mastigophores A); penicilli E (Homotrichs; Holotrichs, p-mastigophores E) see table 2 and fig. 3.

Biological interactions: This genus appears to colonise primarily isidid octocorals (bamboo coral), [3]. Previous studies observed this species on the skeletons of several living isidids [16] as well as on several primnoids. More investigations are necessary to determine if this species has a host preference. It is also not clear if this species is colonizing branches free of tissue, or if it parasitises or competes with the host tissue as it spreads along the branches. Comparison with the closely related genus *Savalia* suggests a parasitic/competitive relationship, but further studies are necessary to understand the ecological relations between this zoanthid and the organisms used as host. Occasionally, colonies of *Clavularia grandiflora* can colonise *Ku. haumeaae* [3]. As found in the genus *Savalia*, a parasitic ascothoracic cirriped was found in several polyps. However, the exact nature of the interactions between this zoanthid and the crustacean are not known. Due to the depths at which this species occurs the absence of observation of zooxanthellae in the histological analyses was not surprising, although the recent findings of *Symbiodinium* in Hawaiian black corals down to 396 m depth [60] indicate we can not exclude the possibility of an extremely low density of symbionts. Attempts to PCR-amplify zooxanthellae using dinoflagellate specific primers and to grow dinoflagellate cultures using fresh material of this species following collections in 2004 were also unsuccessful (A. Baco and R. Gast unpubl data).

Distribution: Throughout the Hawaiian Archipelago seamount and island slopes between 343–575 m on hard substrata in low-sediment areas with high relief. Usually one of the most abundant taxa in this depth range and habitat type, often with other corals present. Also observed in Line and Jarvis Islands, Palmyra Atoll and Kingman Reef however at lower densities [3]. Similar specimens with golden or dark brown, almost black axis, also referred as “*Gerardia*”, were collected in the West Atlantic [16], [17], and in New Zealand [61]. If those zoanthids appear to be closely related enough to be congeneric, species-level relationships require further morphological and molecular analyses.

*Zibrowius* gen. n.

urn:lsid:zoobank.org:act:B139CFB3-E2A3-48D2-A753-84D25645BFA2

Type species: *Zibrowius ammophilus* sp. n.

Etymology: This masculine genus name is dedicated to Dr. Helmut Zibrowius for his precious contribution to deep-sea zoanthid science through the collection of several important samples. He also introduced the first author to deep-sea zoanthid research and especially to octocoral-associated zoanthids such as *Savalia* through his accurate naturalistic observations and critical comments. This genus should not be confused with the coral-parasitic crustacean genus *Zibrowia* Grygier, 1985.

Diagnosis: sand incrusted, arborescent fan shaped colonies, golden skeleton, well developed coenenchyme completely covering the host, can be confused with *Kulamanamana*, but easily distinguished by the presence of sand incrustation in the ectoderm, characteristic insertion/deletion pattern in the 16S V5 region sensu Sinniger et al. [21] (fig. 2).

*Zibrowius ammophilus* sp. n.

Figs. 1 B, 3 (second from top)

urn:lsid:zoobank.org:act:0838CA17-871D-41CA-BFE2-9E17E2318F4B

Etymology: comes from the greek word for sand: ammos, and the greek word for fondness: phileo.

Material examined:

Holotype: P5-589, Cross Seamount (18°43.995′-18°43.759′ N, 158°15.743′-158°15.413′ W), Hawaii, 10.10.2004, 389 m, coll. ARB, colony of 150 polyps of different sizes, USNM 1190191 (formalin-fixed), several fragments, all of them bearing 50 polyps, USNM 1190192 (ethanol-fixed), fragment of 50 polyps MNHG-INVE-82281 (formalin-fixed)

Paratypes: P5-587, Cross Seamount, Hawaii, 08.10.2004, 409 m, coll. ARB, three fragments, all of them bearing 120 polyps of different sizes, BPBM-D2252 (formalin-fixed), twelve fragments, all of them bearing 100 polyps of different sizes, MMC-T5 (formalin-fixed).

Sequences: 18S: KC218407, KC218408; 16S: KC218438, KC218439; COI: KC218394, KC218395.

Diagnosis: Golden axis, orange/brownish colour and presence of mineral incrustations are distinctive characters of this species. In terms of cnidome, the presence of medium to big penicilli E in filaments and body wall appears to be characteristic of this species.

External morphology: In preserved specimens, size of contracted and semi-contracted polyps from 1–5 mm length and 1–3 mm diameter. 20–33 pointed tentacles. In expanded living polyps, length of the tentacles smaller than the diameter of the oral disc. 13–17 bractea (corresponding to capitular ridges on closed polyps) on the edge of the oral disc. Apex of closed polyps flat or slightly rounded. Column cylindrical, with incrusted particles (mainly basalt and foraminifera tests). Well developed coenenchyme completely covering the host. Polyp density variable within the colony with coenenchyme barely visible in the most dense regions (usually the smallest branches) and polyps separated by over 1 cm in the less dense area (such as the main stem). Size of the fan-shaped colonies up to about 50 cm diameter and 70 cm height. Golden scleroproteic skeleton, distinct from the black skeleton found in the genus *Savalia* Nardo 1844 ( = *Gerardia* L.-D. 1864). However, due to the rarity of the species, we could not determine if this zoanthid secretes its own skeleton. As observed in other epizoic genera (*Savalia* and *Antipathozoanthus* particularly) the colony can spread on other substrata at the base of the original host.

Colour: In vivo, polyps, tentacles and coenenchyme are brownish-yellow, similar to the Hawaiian gold coral *Ku. haumeaae*, but distinct enough in colour to be recognised as a different species from the submersible, (fig. 1b).

Microanatomy: 22–32 poorly developed mesenteries following macrocnemic arrangement. Mesoglea in the column very well developed (0.3–0.5 mm), with presence of numerous lacunae with organic content. No retractor or parietobasilar muscles were noticed. All specimens analysed were fertile. Small siphonoglyph, not specially developed. Endodermal sphincter, short and concentrated in the upper part of the column. No zooxanthellae (see previous species for consideration on potential rare symbiosis with zooxanthellae in deep-sea anthozoans).

Cnidome: spirocysts; spirulae (b-mastigophore); penicilli A (p-mastigophores); penicilli E (p-mastigophores or even holotrichs or homotrichs for other authors); homotrich (holotrich for other authors), see table 2 and fig. 3.

Biological interactions: No information could be obtained on organisms (such as octocoral) potentially used as host. Based on external similarities with *Ku. haumeaae* and *Savalia* spp, this species might colonise octocorals.

Distribution: To date, only observed or collected on Cross Seamount.

*Hurlizoanthus* gen. n.

urn:lsid:zoobank.org:act:C0034FA0-804C-476C-B67A-F79DF0755DD0

Type species: *Hurlizoanthus parrishi* sp. n.

Etymology: The genus name refers to HURL (the Hawaiian Undersea Research Laboratory) which has provided significant funding and the use of their submersible *Pisces* for deep-sea coral research in the Hawaiian Archipelago. Work with agency has provided many deep-sea coral species new to science. Due to budget restrictions HURL was closed in the year of the description of these zoanthid species and this genus name is to acknowledge the important contribution of HURL in the discovery of deep-sea diversity around the Hawaiian archipelago. The ending -zoanthus, is a common ending of genera names in the order, historically referring to the flower-like appearance of the animal polyps.

Diagnosis: Macrocnemic genus associated with primnoids. Characteristic insertion/deletion pattern in the 16S V5 region sensu Sinniger et al. [21] (fig. 2).

*Hurlizoanthus parrishi* sp. n.

Figs. 1 C, 3 (third from top)

urn:lsid:zoobank.org:act:F3CF4097-6439-4E8B-A154-083CD36F5978

Etymology: This species name is dedicated to Dr. Frank Parrish for his contribution to the knowledge of the biology of *Ku. haumeaae* and other precious corals in the Hawaiian Archipelago as well as his support of the research of ARB and FS.

Material examined:

Holotype: P5-595, Keahole Point (19°47.907′-19°48.418′ N, 156°07.786′-156°07.631′ W), Hawaii, 17.10.2004, 390 m, coll. ARB, ramified colony, more than 300 polyps, USNM 1190193 (formalin-fixed), branches of the main colony, 70–80 polyps, USNM 1190194 (ethanol-fixed).

Paratypes: P5-533, NW Laysan Island, Hawaii, 19.10.2003, 496 m, coll. ARB, well developed colony, more than 400 polyps, NMST-Co 1549 (ethanol-fixed), small branches with about 70 polyps, NIWA-84100 (ethanol-fixed), several branches bearing 150 polyps, BPBM-D2253 (ethanol-fixed).

Sequences: 18S: KC218402; 16S: KC218433; COI: KC218389.

Diagnosis: White colour and few ramifications are distinctive characters of this species in the studied area. In the cnidome, the presence of big penicilli E ( = Holotrichs; Homotrichs; p-mastigophores E) in the body wall appears to be characteristic of this species..

External morphology: In preserved specimens, size of contracted and semi-contracted polyps from 1–5 mm length and 1–3 mm diameter. 30–35 pointed tentacles. In expanded living polyps, length of the tentacles shorter than the diameter of the oral disc. 15–17 bractea (corresponding to capitular ridges on closed polyps) on the edge of the oral disc. Apex of closed polyps flat. Column cylindrical, with incrusted particles (foraminifer tests and basalt). Polyps arranged around the axis, usually in an orthogonal arrangement. Thin coenenchyme completely covering the host. Coenenchyme always visible between the polyps. Colony size reaching 30 cm height and 15 cm diameter, with no additional branching apparent beyond the host octocoral skeleton.

Colour: In vivo, polyps, tentacles and coenenchyme are white (fig. 1c). Upon formalin fixation tissues turn light red as observed in several other zoanthid species.

Microanatomy: 20 to 30 mesenteries following the macrocnemic arrangement. Most mesenteries fertile, including incomplete mesenteries. Mesoglea of the column 0.1–0.2 mm thick, showing sometimes a line of lacunae with organic content. No retractor muscle or parietobasilar muscle features were noticed. Small single siphonoglyph. Short endodermal sphincter, concentrated in the upper part of the column, forming processes (as can be observed in *I. primnoidus* Carreiro-Silva et al. 2010 but see discussion). No zooxanthellae.

Cnidome: spirocysts; spirulae (b-mastigophore); penicilli A (p-mastigophore); penicilli (p-mastigophore); penicilli E (p-mastigophore or even holotrich or homotrich for other authors); homotrich (holotrich for other authors), see table 2 and fig. 3.

Biological interactions: Based on the observation of the axis branching and aspect, this genus colonises unidentified octocorals (Primnoidae).

Distribution: Besides collection locations, also observed in submersible and ROV videos at the Makapu'u coral bed off Oahu, Hawaii.

*Kauluzoanthus* gen. n.

urn:lsid:zoobank.org:act:38ACC0FC-80F9-483B-B4F6-9C35A5287F39

Type species: *Kauluzoanthus kerbyi* sp. n.

Etymology: This genus is named after Kaulu, a trickster god that killed Haumea (see etymology of *Kulamanamana haumeaae*), and -zoanthus, a common ending of genera names in the order, historically referring to the flower-like appearance of the animal polyps.

Diagnosis: Characteristic insertion/deletion pattern in the 16S V5 region sensu Sinniger et al. [21] (fig. 2). Polyps not contracting when fixed (fig. 1d).

*Kauluzoanthus kerbyi* sp. n.

Figs. 1 E, 3 (second from bottom)

urn:lsid:zoobank.org:act:6090DA23-F323-45A0-A253-19881C2865DE

Etymology: this species is named after Terry Kerby, who has been a HURL submersible pilot through most of the time period HURL has been in operation and was pilot during the collection of most of the specimens in this paper. His knowledge of the Hawaiian deep-sea fauna considerably facilitated the sampling of the zoanthids described here.

Material examined:

Holotype: P5-593, Keahole Point (19°48.203′-19°47.943′ N, 156°08.047′-156°07.478′ W), Hawaii, 15.10.2004, 397 m, coll. ARB, 30 polyps of different sizes, USNM 1190195 (formalin-fixed), 20–25 polyps of different sizes, USNM 1190196 (ethanol-fixed).

Paratypes: P5-590, Cross Seamount, Hawaii, 11.10.2004, 343 m, coll. ARB, 35 polyps with specially developed column, some polyps of *Ku. haumeaae* also present in the sample, NMST-Co 1550 (formalin-fixed), 5 polyps, NMST-Co 1551 (ethanol-fixed); P5-523, Bank 8, Hawaii, 06.10.2003, 536 m, 10 polyps, BPBM-D2254 (ethanol-fixed). All samples collected by ARB.

Sequences: 18S: KC218404, KC218405, KC218406; 16S: KC218435, KC218436 KC218437; COI: KC218391, KC218392, KC218393.

Diagnosis: Light beige polyps, large polyps, associated to Ku. haumeaae. In the cnidome, the presence of medium to big penicilli E in the tentacles and body wall and special spirulae in the tentacles appears to be characteristic of this species.

External morphology: In preserved specimens, size of polyps from 1–5 mm length and 1–5 mm diameter (although some colonies may have very short polyps). 23–28 pointed tentacles. In fixed polyps, length of the tentacles up to the diameter of the oral disc. No bractea or marginal teeth observed may be present but inconspicuous to be observed under dissecting microscope. Column cylindrical, no incrusted particles. Thin developed coenenchyme partially covering *Kulamanamana haumeaae* colonies as well as several octocorals, forming patches up to several centimetres diameter. So far, no host colony has been observed that was fully covered by this zoanthid.

Colour: In vivo, polyps, tentacles and coenenchyme light beige.

Microanatomy: 20–25 mesenteries following the macrocnemic arrangement. No gonads observed. Due to the short length of the column, mesenteries are very difficult to observe. Mesenterial filaments well developed. Deep and narrow siphonoglyph. No sphincter observed. Retractor muscle and parietobasilar muscles with very low development. Tentacle musculature with ectodermal process. No zooxanthellae.

Cnidome: spirocysts, spirulae (b-mastigophore); special spirulae (special b-mastigophores for other authors), penicilli A (p-mastigophore); penicilli E (p-mastigophore or even holotrich or homotrich for other authors); homotrich (holotrich for other authors), see table 2 and fig. 3. Cellular structures containing potential micronematocysts were observed in the body wall.

Biological interactions: This species was found on *Kulamanamana haumeaae* colonies. Further investigations are necessary to study the range of hosts used by this species.

Distribution: So far found in the Hawaiian archipelago with a distribution similar to the Hawaiian gold coral, *Ku. haumeaae*. Similar and potentially identical zoanthid was observed in other Pacific locations such as Line, Jarvis, Palmyra and Kingman [3].

*Bullagummizoanthus* gen. n.

urn:lsid:zoobank.org:act:8CDA7F91-217A-48CA-B855-90B6B4BD1E3D

Type species: *B. emilyacadiaarum* n. sp.

Etymology: the name refers to the affinity of this zoanthid genus to bubble gum corals (family Paragorgiidae) with “bulla” and “gummi” being the latin words for “bubble” and “gum” respectively, and -zoanthus, a common ending of genera names in the order, historically referring to the flower-like appearance of the animal polyps.

Diagnosis: Characteristic insertion/deletion pattern in the 16S V5 region sensu Sinniger et al. [21] (fig. 2).

*Bullagummizoanthus emilyacadiaarum* sp. n.

Figs. 1 F, 3 (bottom)

urn:lsid:zoobank.org:act:35C895F6-CA66-4189-BD77-487749DA25A3

Etymology: This species is dedicated to Emily and Acadia Baco-Taylor, both born during the taxonomical investigations of these Hawaiian species and presenting positive taxis towards bubble gum.

Material examined:

Holotype: P5-595, Keahole Point (19°47.907′-19°48.418′ N, 156°07.786′-156°07.631′ W), Hawaii, 17.10.2004, 558 m, coll. ARB, fragment of 70 polyps, USNM 1190197 (formalin-fixed), fragment of 32 polyps, USNM 1190198 (ethanol-fixed), fragment of 25 polyps, BPBM-D2255 (formalin-fixed), fragment of 35 polyps, MMC-T6 (formalin-fixed).

Sequences: 18S: KC218403; 16S: KC218434; COI: KC218390.

Diagnosis: colony distributed across entire host colony, but not entirely covering its host, low density of polyps, associated with a paragorgiid. In the cnidome, the presence of special spirulae in the body wall and special penicilli E in the filaments appears to be characteristic of this species.

External morphology: In preserved specimens, size of contracted and semi-contracted polyps from 1–7 mm length and 2–7 mm diameter. 30–39 pointed tentacles. 15–20 bractea (corresponding to capitular ridges on closed polyps) on the edge of the oral disc. Apex of closed polyps flat. Column cylindrical, with incrusted mineral particles (white sand grains and gorgonian sclerites). Poorly developed coenenchyme not completely covering the host. Polyp sparsely distributed on the host, often in groups of few joined polyps issued of asexual reproduction (fig. 1e).

Size of the colony observed at least 30–40 cm diameter.

Colour: In situ, polyps bright yellow, in strong contrast to bright red of host. In vivo, polyps, tentacles and coenenchyme beige, colour influenced by incrustation, especially pink sclerites. No significant change of colour in formalin.

Microanatomy: 30 to 40 mesenteries following the macrocnemic arrangement. Most mesenteries fertile. No retractor muscle or parietobasilar muscle features were noticed. Single conspicuous siphonoglyph. Weak endodermal sphincter, difficult to observe in small polyps, extending over the first half of the column. No sinus but small processes were observed. No zooxanthellae.

Cnidome: spirocysts; spirulae (b-mastigophore); special spirulae (special b-mastigophores for other authors); penicilli A (p-mastigophore); penicilli E (p-mastigophore or even holotrich or homotrich for other authors); special penicilli E (new category of nematocysts, enlarged and incurved capsule with a thick filament full of conspicuous spines inside); homotrich (holotrich for other authors). Sporadic special spirulae(special b-mastigophores for other authors) (not shown). See table 2 and fig. 3.

Biological interactions: This species colonises a paragorgiid coral, most likely *Paragorgia coralloides*. Due to the coexistence of the octocoral and the zoanthid, further studies are necessary to determine the type of relationship between the two organisms.

Distribution: The single colony observed was sub-sampled for this description. However, similar zoanthids are frequently observed on samples of *Paragorgia coralloides* throughout the Pacific, further investigation on such specimens are required to confirm the identity between this specimen and these other zoanthids.

Remark on the diagnoses: Diagnoses and descriptions were based on single species for each genera, moreover originating from a restricted locality. Therefore, it is likely that those diagnoses will be completed and modified with the discovery of related species around the world, as happened for the genera *Mesozoanthus* and *Abyssoanthus* [8], [9], [62].

### Phylogenetic results

**Figure 4:**
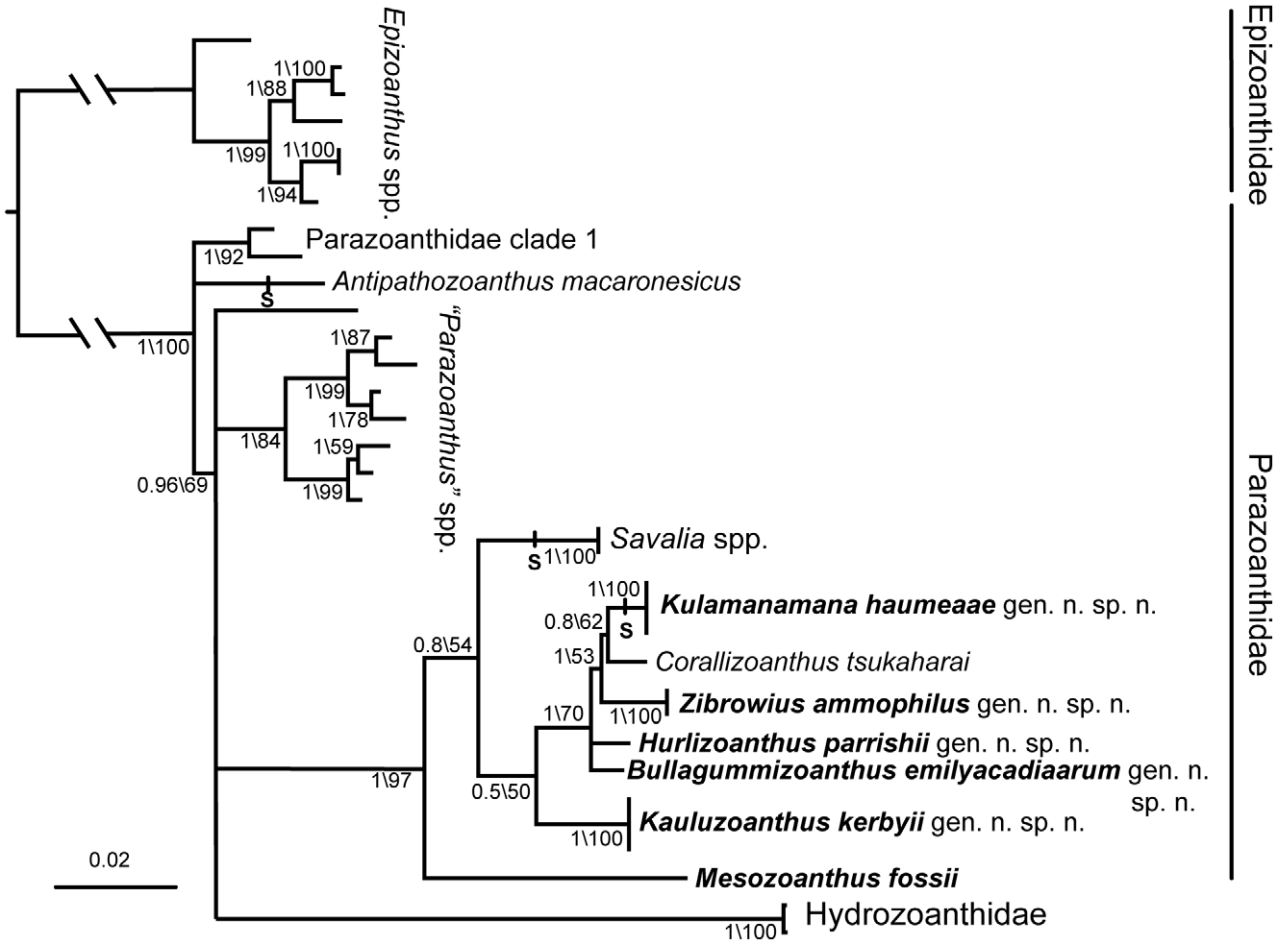
Phylogenetic tree of the Hawaiian gold coral (*Ku. haumeaae*) and related zoanthids. Bayesian tree based on concatenated 18S, COI and 16S genes. Values at the nodes represent posterior probabilities and bootstrap values respectively. “**S**” indicate the ability to secrete a skeleton. Values below posterior probabilities of 0.5 or 50% bootstrap were considered as unresolved. Hawaiian zoanthids described here are indicated in bold. Epizoanthidae are used as outgroup. Vertical bars indicate the species belonging to Epizoanthidae and Parazoanthidae respectively.

Maximum Likelihood and Bayesian trees topologies are similar (fig. 4), clearly separating the different genera described here. In comparison to the Bayesian tree shown in fig. 4, ML trees (not shown) place *H. parrishi* as sister group of the *Z. ammophilus*, *Ku. haumeaae*, *C. tsukaharai* group (ML bootstrap support: 56) and isolate “Parazoanthidae clade 1” at the base of all other Parazoanthidae and Hydrozoanthidae (those groups forming a monophyly supported by a ML bootstrap support of 62). The information provided by three markers used distinguishes most specimens at the species level. At family level, the results confirmed the hypotheses published earlier [7], [21] with the family Hydrozoanthidae and the Brachycnemina (not shown) diverging from the Parazoanthidae. However, the phylogenetic relationships among Parazoanthidae are mostly unresolved and no reliable information could be obtained in regard to the presumed close relation between the genus *Antipathozoanthus* and the octocoral-associated zoanthids [22]. Within parazoanthids, the monophyly of all octocoral-associated zoanthids is supported. Both analyses place *Mesozoanthus fossii*, another parazoanthid not associated to specific organisms, as sister group of this clade and *Savalia* species branch at the base of the octocoral-associated clade. *Ka. kerbyi*, whose ecological relation to octocorals or octocoral-associated zoanthids are unclear, appears distinct from the unresolved clade formed by the remaining octocoral-associated zoanthids. However, it must be noted that the basal position of *Savalia* in relation with the position of *Ka. kerbyi* have weak support in the statistical analyses. Within the clade grouping the other octocoral-associated zoanthids, while ML analyses do not show supported evidence of evolutionary relationships, Bayesian analyses separate *Ku. haumeaae* and *C. tsukaharai* as a sister clade to *Z. ammophilus* and distinct from *H. parrishi* and *B. emilyacadiaarum*.

## Discussion

### How to classify these new species?

Based on the phylogenetic trees, several options could be considered to define the taxonomy of Hawaiian deep octocoral-related zoanthids.

1. The Hawaiian gold coral, described here as *Ku. haumeaae*, has historically been referred as “*Gerardia* sp.”, a younger synonym of *Savalia* Nardo 1844. Based on the close molecular distances between all octocoral-associated zoanthids, all species examined could have been moved to the genus *Savalia* which would then also include the genus *Corallizoanthus*. However, *Savalia* is characterised by its ability to secrete a skeleton and this ability is not shared by *Corallizoanthus*, *H. parrishi* and *Ka. kerbyi* (unclear in *H. ammophilus*). Morphology of the colonies is completely different between those species, with *Corallizoanthus* colonies consisting of individual polyps connected together only temporarily after polyp division, *B. emilyacadiaarum* also presents a poorly developed coenenchyme and groups of polyps scattered along the host, and other species usually present dense colonies with well developed coenenchyme covering most of the host.
2. Strictly based on phylogenetic tree information, Hawaiian species might be placed in the genus *Corallizoanthus*, however, the remarks mentioned above apply also in this situation and while *Savalia* would remain independent, the secretion of a skeleton by *Ku. haumeaae* appears as a character difficult to ignore when grouping this species with non-skeleton secreting zoanthids.
3. Ignoring phylogenetic information at genus level and based on the history of referring to the Hawaiian gold coral as *Gerardia* sp., all the skeleton secreting zoanthids could be grouped into *Savalia*. However, the taxonomic significance of the skeleton is unknown as well as information on biochemical similarities between secretions of *Savalia* and *Ku. haumeaae* is still missing. The absence of sand incrustation in *Ku. haumeaae* is at least as important character to separate *Kulamanamana* from *Savalia* as is the skeleton to group those species together.
4. Each new species, sharing only a few characteristics and possessing several unique features can be placed in their own genus. The main issue of this solution is the creation of numerous monospecific genera. Nevertheless, in such a restricted and isolated geographic location as was investigated in this study, it is not unlikely to find genera represented only by a single species. The situation is also similar to *Mesozoanthus* or *Abyssoanthus*, monospecific when first described, but since then new species have been discovered around the world [7]–[9], [62].

Based on our knowledge on zoanthid taxonomy and evolution, the hypothesis of the Hawaiian zoanthids examined in this study belonging to 5 genera was retained and is presented here.

### Molecular morphology

As mentioned above, morphological characters are sometimes confusing and can be difficult to compare between different authors. Two of the molecular markers used in this study are ribosomal gene fragments. This implies that the DNA sequence will not be translated into a protein, but the sequence of the RNA transcripted from the DNA will directly define the shape of this RNA strand to form the ribosome. The mitochondrial 16S rDNA is particularly interesting for DNA-based taxonomy as most of the variation appears under the form of insertion and deletion of relatively short strings of DNA. Reconstruction of the natural shape of the ribosome based on the sequence is extremely difficult as it is dependent on several external factors, such as temperature and interactions with other molecules among others. Insertion and deletion events are clear signs of ribosome morphological variation as they are directly related to loops or hairpin folding of the RNA. Our results show that the V5 region (sensu Sinniger et al. [21]) can be used to distinguish zoanthids at the generic level (fig. 2). The insertion/deletion pattern of the V5 region could then be considered as the meeting of molecular and morphological characteristics in zoanthids.

### *Isozoanthus primnoidus* and *H. parrishi* two primnoid-associated zoanthids

Recently Carreiro-Silva et al. [28] described *Isozoanthus primnoidus* from Azores, a zoanthid ecologically similar to *H. parrishi*. While unfortunately no DNA information was published or could be obtained from the type specimens of this new species, the morphological similarities and potential placement of *H. parrishi* within the genus *Isozoanthus* must be addressed here. *I. primnoidus* was assigned to the genus *Isozoanthus* based on sphincter characteristics and absence of several histological structures. The same characters have been used in the description of *Hydrozoanthus antumbrosus* (originally described as *Isozoanthus*), however, *Isozoanthus* as interpreted recently is likely a “catch all” genus for unrelated species, this statement was based on the comparison with *I. sulcatus*, a widely accepted *Isozoanthus* species, showing clear divergences from *H. antumbrosus* [7]. This statement was further supported by the by the examination of the type species of the genus *Isozoanthus*, *I. gigantus* [29]. This species also appears completely divergent to any of the zoanthids described here (Fig. S1). The ecology of the original species of *Isozoanthus* also supports the absence of a relationship between the species here, *I. primnoidus* (associated with primnoid octocorals) and formerly described *Isozoanthus* species, encrusting rocks or in some cases hexactinellid stalks. Marked morphological differences between *I. primnoidus* and other *Isozoanthus* species are also mentionned in the original description [28]. Preliminary molecular results (not shown) obtained from potential *I. primnoidus* from Mediterranean Sea support a clear distinction between *I. primnoidus* and *H. parrishi*.

### Skeleton-secreting zoanthids

The molecular divergence observed between *Ku. haumeaae* and *Savalia* spp. is surprising considering the absence of variation observed in other samples of *Savalia* collected worldwide and the apparent morphological similarities between those zoanthids. Moreover the presence of zoanthids not secreting a skeleton within the monophyletic group comprising *Savalia* and *Ku. haumeaae* would suggest either a loss or multiple acquisitions of this very particular ability during the evolution of this group. The potential secretion of a skeleton in *Z. ammophilus* remains to be examined in detail, so no conclusion can be made on this species at the moment. All these zoanthids belong to the same monophyletic clade associated with octocorals, however the potential secretion of a skeleton in the genus *Antipathozoanthus*, a genus often associated with antipatharians and placed outside of this large octocoral associated monophyletic group requires further investigation [22], [63] but see [7], [21]. The confirmation of skeleton secretion in *Antipathozoanthus* would strongly support the hypothesis of multiple evolution of the capacity to build a scleroproteic axis among zoanthids.

### “Alien” nematocysts?

The presence of nematocysts types characteristic of medusozoa, such as rounded “special” spirulae [64], [65] in *Z. ammophilus* and mainly in *B. emilyacadiaarum* could originate from the hypothesized feeding habits of these species. Carlgren [23] discussed this issue in *Epizoanthus incrustatus* and *E. erdmanni* concluding, based on the heterogeneity between samples of the same species, a probable external origin of medusozoan nematocysts types. However, until further clarification of the trophic behaviour of these zoanthids we decided to include those nematocysts as part of the cnidome equipment of the zoanthids. This decision is supported by the finding of similar nematocysts categories in other zoanthids [63].

### Habitat and ecology

All those zoanthids were found in areas of seamount pinnacles or on the slope of Hawaiian Islands exposed to currents. This habitat was frequently shared with other precious corals such a *Corallium lauuense*, other octocorals and antipatharians, as described in detail for *Ku. haumeaae* in previous studies [3]–[5]. However, the distribution of those epizoic species is expected to be highly dependent on the distribution of the host organisms.

Molecular phylogeny supports some relationships between the evolution of the order and the group of organisms used as host. However, for species such as *Ku. haumeaae*, the invasive and destructive nature of investigations necessary to identify the original host organism limits the number of samples examined. Further studies are necessary to determine host specificity in these zoanthids as well as on the nature of the interaction between the zoanthids and their host. However, due to the slow growth of the colonies and the invasive nature of current methods to investigate the substrate host in adult colonies, caution must be taken not to impact the stocks of these vulnerable species.

## Conclusion

### Deep-sea diversity

The zoanthid diversity found in the deeper waters surrounding Hawaii is exceptional in comparison to the diversity found on coral reef in the same area. 7 species of zoanthids belonging to the 3 genera usually found in coral reefs (*Palythoa*, *Zoanthus* and *Isaurus*) were identified in Hawaii [66]. Tropical coral reefs are commonly assumed to be hot spots of biological diversity, however, the discovery of such species and genera diversity in octocoral-associated zoanthids at depth ranging from 300–500 meters would suggest an even higher biodiversity in subphotic coral communities along the steep slopes of oceanic islands and seamounts. A similar pattern is observed for octocorals in the Hawaiian Archipelago, with the vast majority of known Hawaiian octocoral species occurring only in deep water [67]. In addition to the epizoic zoanthids examined in this study, at least three other non epizoic, undescribed species, two belonging to the genus *Epizoanthus* and one probable Parazoanthidae were found in the same habitat. This discovery illustrates the amount of zoanthid diversity that remains to be discovered on deep-sea hard substrate environments. Understanding the speciation processes and distribution of these zoanthids will certainly contribute to a better understanding of the past oceanographic patterns in the worldwide oceans as well as contributing to developing efficient impact assessment methods in relation to deep-sea resource exploitation.

## Supporting Information

Table S1 Sequences information. n/a = not available. FS refers to the sample number in the first author collection. Due to the short length of the sequences or availability, sequences of *Microzoanthus* and *Isozoanthus* were used only in the 16S analyses presented in the fig. S1.
(DOC)

Figure S1 **Phylogenetic tree based on 16S including** ***Microzoanthus*** **and** ***Isozoanthus*** **.** ML phylogenetic tree reconstructed using the partial 16S sequences. The position of *Isozoanthus gigantus* near *Epizoanthus* is well supported. ML bootstrap values are indicated at the nodes. Only the groups supported by 50% or more bootstrap are represented. Microzoanthidae are used as outgroup.
(PDF)

## References

[1] Miller K, Neil H, Tracey D (2009) Recent advances in deep-sea coral science and emerging links to conservation and management of deep-sea ecosystems. Mar Ecol Prog Ser 397: 1–5.

[2] Krieger KJ, Wing BL (2002) Megafauna associated with deepwater (*Primnoa* spp.) in the Gulf of Alaska. Hydrobiologia 471: 83–90.

[3] Parrish FA, Baco A (2007) State of Deep Coral Ecosystems in the United States Western Pacific Region: Hawaii and the United States Pacific Islands. In: Lumsden SE, Hourigan TF, et al.., editors. The State of Deep Coral Ecosystems of the United States. NOAA Technical Memorandum CRCP-3. Silver Spring, MD: National Oceanic and Atmospheric Administration. pp. 155–194.

[4] Baco AR (2007) Exploration for deep-sea corals on North Pacific seamounts and islands. Oceanography 20 (4) 108–117.

[5] Parrish FA (2006) Precious corals and subphotic fish assemblages. Atoll Res Bull 543: 425–438.

[6] Muirhead A, Tyler PA, Thurston MH (1986) Reproductive biology and growth of the genus *Epizoanthus* (Zoanthidea) from the north-east Atlantic. J Mar Biol Assoc UK 66: 131–143.

[7] Sinniger F, Reimer JD, Pawlowski J (2010) The Parazoanthidae (Hexacorallia: Zoantharia) DNA taxonomy: Description of two new genera. Mar Biodiv 40: 57–70.

[8] Reimer JD, Sinniger F (2010) Discovery and description of a new species of *Abyssoanthus* (Zoantharia: Hexacorallia) at the Japan Trench: the world's deepest known zoanthid. Cah Biol Mar 51 (4) 451–457.

[9] Sinniger F, Zelnio K, Taviani M, Reimer JD (2010) Presence of *Abyssoanthus* sp. (Anthozoa: Zoantharia) in the Mediterranean Sea: indication of a new cold seep or of ecological tolerance of *Abyssoanthus* to non-chemotrophic environments? Cah Biol Mar 51 (4) 475–478.

[10] Grigg RW (1974) Distribution and abundance of precious corals in Hawaii. Proc 2nd Int Coral Reef Symp, Brisbane 235–240.

[11] Grigg RW (2002) Precious corals in Hawaii: discovery of a new bed and revised management measures for existing beds. Mar Fish Rev 64: 13–20.

[12] Marschal C, Garrabou J, Harmelin JG, Pichon M (2004) A new method for measuring growth and age in the precious red coral *Corallium rubrum* (L.). Coral Reefs 23: 423–432.

[13] Roark EB, Guilderson TP, Dunbar RB, Ingram BL (2006) Radiocarbon-based ages and growth rates of Hawaiian deep-sea corals. Mar Ecol Prog Ser 327: 1–14.

[14] Roark EB, Guilderson TP, Dunbar RB, Fallon SJ, Shester GS, et al (2009) Extreme longevity in proteinaceous deep-sea corals. Proc Natl Acad Sci USA 106: 5204–5208. 19307564 10.1073/pnas.0810875106 PMC2663997

[15] Parrish FA, Roark EB (2009) Growth validation of gold coral *Gerardia* sp. in the Hawaiian Archipelago. Mar Ecol Prog Ser 397: 163–172.

[16] Goodfriend GA (1997) Aspartic acid racemization and amino acid composition of the organic endoskeleton of the deep-water colonial anemone *Gerardia*: determination of longevity from kinetic experiments. Geochim Cosmochim Acta 61: 1931–1939.

[17] Druffel ERM, Griffin S, Witter A, Nelson E, Southon J, et al (1995) *Gerardia*: Bristlecone pine of the deep-sea? Geochim Cosmochim Acta 59: 5031–5036.

[18] Grigg RW (1976) Fishery management of precious and stony corals in Hawaii. Report No. UNIHI-SEAGRANT-TR-77-03. Honolulu, HI: University of Hawaii Sea Grant Program.

[19] Poche F (1914) Das System der Coelenterata. Arch Naturgesch 80 (5) 47–128.

[20] Häussermann V (2003) Zoanthidea. In: Hofrichter, R. (ed.), Das Mittelmeer, bestimmungsführer Band II/1. Heidelberg: Spektrum Akad. Verl. pp. 501–505.

[21] Sinniger F, Montoya-Burgos JI, Chevaldonné P, Pawlowski J (2005) Phylogeny of the order Zoantharia (Anthozoa, Hexacorallia) based on the mitochondrial ribosomal genes. Mar Biol 147: 1121–1128.

[22] Ocaña O, Brito A, Gonzalez G, Herrera R (2007) Additions in relation to Gerardiidae from the Macaronesian waters and the Mediterranean Sea (Anthozoa: Zoantharia). Vieraea 35: 163–168.

[23] Carlgren O (1913) Zoantharia. The Danish Ingolf Expedition 5 (4) 1–65.

[24] Lwowsky F (1913) Revision der Gattung *Sidisia* Gray (Epizoanthus auct.). Ein Beitrag zur Kenntnis der Zoanthiden. Zool Jb Abt f Syst 34: 557–614.

[25] Sanamyan NP, Sanamyan KE (2007) Deep-water Actiniaria from East Pacific hydrothermal vents and cold seeps. Invert Zool 4: 83–102.

[26] Swain TD (2009) *Isozoanthus antumbrosus*, a new species of zoanthid (Cnidaria: Anthozoa: Zoanthidea) symbiotic with Hydrozoa from the Caribbean, with a key to hydroid and sponge-symbiotic zoanthid species. Zootaxa 2051: 41–48.

[27] Ritt B, Sarrazin J, Caprais J-C, Noël P, Gauthier O, et al (2010) First insights into the structure and environmental setting of cold-seep communities in the Marmara Sea. Deep-Sea Res Pt I 57: 1120–1136.

[28] Carreiro-Silva M, Braga-Henriques A, Sampaio I, de Matos V, Porteiro FM, et al (2010) *Isozoanthus primnoidus*, a new species of zoanthid (Cnidaria: Zoantharia) associated with the gorgonian *Callogorgia verticillata* (Cnidaria: Alcyonacea). ICES J Mar Sci doi:10.1093/icesjms/fsq073.

[29] Swain TD (2010) Evolutionary transitions in symbioses: dramatic reductions in bathymetric and geographic ranges of Zoanthidea coincide with loss of symbioses with invertebrates. Mol Ecol 19: 2587–2598. 20497327 10.1111/j.1365-294X.2010.04672.x

[30] Sinniger F, Häussermann V (2009) Zoanthids (Cnidaria: Hexacorallia: Zoantharia) from shallow waters of the southern Chilean fjord region, with descriptions of a new genus and two new species. Org. Div. Evol 9: 23–36.

[31] Fujii T, Reimer JD (2011) Phylogeny of the highly divergent zoanthid family Microzoanthidae (Anthozoa, Hexacorallia) from the Pacific. Zool. Scr 40: 418–431.

[32] Ryland JS, Brasseur MM, Lancaster JE (2004) Use of cnidae in taxonomy: implications from a study of *Acrozoanthus australiae* (Hexacorallia, Zoanthidea). J Nat. Hist 38: 1193–1223.

[33] Reimer JD, Ono S, Iwama A, Takishita K, Tsukahara J, et al (2006) Morphological and molecular revision of *Zoanthus* (Anthozoa: Hexacorallia) from southwestern Japan, with descriptions of two new species. Zool Sci 23: 261–275. 16603820 10.2108/zsj.23.261

[34] Reimer JD, Hirano S, Fujiwara Y, Sinniger F, Maruyama T (2007) Morphological and molecular characterization of *Abyssoanthus nankaiensis*, a new family, new genus and new species of deep sea zoanthid (Anthozoa: Hexacorallia: Zoantharia) from a northwest Pacific methane cold seep. Invert Syst 21: 255–262.

[35] Reimer JD, Nonaka M, Sinniger F, Iwase F (2008) Morphological and molecular characterization of a new genus and new species of parazoanthid (Anthozoa: Hexacorallia: Zoantharia) associated with Japanese red coral. Coral Reefs 27: 935–949.

[36] Sinniger F, Reimer JD, Pawlowski J (2008) Potential of DNA sequences to identify zoanthids (Cnidaria: Zoantharia). Zool Sci 25: 1253–1260. 19267653 10.2108/zsj.25.1253

[37] Reimer JD, Hirose M, Nishisaka T, Sinniger F, Itani G (2010) *Epizoanthus* spp. Associations Revealed Using DNA Markers: A Case Study from Kochi, Japan. Zool. Sci 27: 729–734. 20822400 10.2108/zsj.27.729

[38] Bertoloni A (1819) Amoenitates Italicae, sistentes Opuscula ad Rem herbariam et zoologiam Italiae spectantia. Bononiae [1]: 472.

[39] Cutress CE, Pequegnat WE (1960) Three new species of Zoantharia from California. Pac Sci 14: 89–100.

[40] Schmidt H (1972) Prodromus zu einer Monographie der mediterranen Aktinien. Zoologica, Stuttgart 121: 1–146.

[41] Hartog JC den (1980) Caribbean shallow water Corallimorpharia. Zool Verh Leiden 176: 1–83.

[42] Hartog JC den, Ocaña O, Brito A (1993) Corallimorpharia collected during the CANCAP expedition (1976–1986) in the south-eastern part of the North Atlantic. Zool. Verh 282: 1–76.

[43] Ryland JS, Lancaster JE (2004) A review of zoanthid nematocyst types and their population structure. Hydrobiologia 530/531: 179–187.

[44] Medlin L, Elwood HJ, Stickel S, Sogin ML (1988) The characterization of enzymatically amplified eukaryotic 16S-like rRNA-coding regions. Gene 71: 491–499. 3224833 10.1016/0378-1119(88)90066-2

[45] Apakupakul K, Siddall ME, Burrelson E (1999) Higher-level relationships of leeches based on morphology and gene sequences. Mol Phyl Evol 12: 350–359. 10.1006/mpev.1999.0639 10413628

[46] Hall TA (1999) BioEdit: a user-friendly biological sequence alignment editor and analysis program for Windows 95/98/NT. Nucleic Acids Symp Ser 41: 95–98.

[47] Jobb G, von Haeseler A, Strimmer K (2004) TREEFINDER: A powerful graphical analysis environment for molecular phylogenetics. BMC Evol Biol 4: 18. 15222900 10.1186/1471-2148-4-18 PMC459214

[48] Ronquist F, Huelsenbeck JP (2003) Bayesian phylogenetic inference under mixed models. Bioinformatics 19: 1572–1574. 12912839 10.1093/bioinformatics/btg180

[49] Grigg RW (1993) Precious coral fisheries of Hawaii and the US. Pacific Islands. Mar Fish Rev 55: 50–60.

[50] Parrish FA, Abernathy K, Marshall GJ, Buhleier BM (2002) Hawaiian monk seals (*Monachus schauinslandi*) foraging in deep-water coral beds. Mar Mammal Sci 18: 244–258.

[51] Baco AR, Shank TM (2005) Population genetic structure of the Hawaiian precious coral *Corallium lauuense* (Octocorallia: Coralliidae) using microsatellites. In: Freiwald A, Roberts JM, editors. Cold-water Corals and Ecosystems. Berlin: Springer-Verlag. pp. 663–678.

[52] Etnoyer P, Morgan LE (2005) Habitat-forming deep-sea corals in the Northeast Pacific Ocean. In: Freiwald A, Roberts JM, editors. Cold-water Corals and Ecosystems. Berlin: Springer-Verlag. pp. 331–343.

[53] Auster PJ (2007) Conservation and adaptive management of seamount and deep-sea coral ecosystems. In: George RY, Cairns SD, editors. Conservation and adaptive management of seamount and deep-sea coral ecosystems. Miami, FL: Rosenstiel School of Marine and Atmospheric Science, University of Miami. pp. 93–99.

[54] Parrish FA (2007) Density and habitat of three deep-sea corals in the lower Hawaiian chain. In: George RY, Cairns SD, editors. Conservation and adaptive management of seamount and deep-sea coral ecosystems. Miami, FL: Rosenstiel School of Marine and Atmospheric Science, University of Miami. pp 185–194.

[55] Cairns SD (2007) Deep-water corals: an overview with special reference to diversity and distribution of deep-water scleractinian corals. Bull Mar Sci 81: 311–322.

[56] Waller RG, Baco AR (2007) Reproductive morphology of three species of deep-water precious corals from the Hawaiian Archipelago: *Gerardia* sp, *Corallium secundum*, and *Corallium lauuense* . Bull Mar Sci 81: 533–542.

[57] Murray Roberts J, Wheeler AJ, Freiwald A, Cairns SD (2009) Cold-Water Corals: The Biology and Geology of Deep-Sea Coral Habitats. Cambridge: Cambridge University Press. 334 p.

[58] Clark MR, Kelley C, Baco A, Rowden A (2011) Fauna of cobalt-rich ferromanganese crust seamounts. International Seabed Authority Tech Study No. 8: 92.

[59] Previati M, Palma M, Bavestrello G, Falugi C, Cerrano C (2010) Reproductive biology of *Parazoanthus axinellae* (Schmidt, 1862) and *Savalia savaglia* (Bertoloni, 1819) (Cnidaria, Zoantharia) from the NW Mediterranean coast. Mar Ecol 31: 555–565.

[60] Wagner D, Pochon X, Irwin L, Toonen RJ, Gates RD (2011) Azooxanthellate? Most Hawaiian black corals contain *Symbiodinium* . Proc Biol Sci 278: 1323–1328. 20961904 10.1098/rspb.2010.1681 PMC3061131

[61] Tracy DM, Anderson OF, Naylor JR (2011) A guide to common deepsea invertebrates in New Zealand waters. New Zealand Aquatic Environment and Biodiversity Report (86) 317.

[62] Reimer JD, Sinniger F (2010) Unexpected diversity in Canadian Pacific zoanthids (Cnidaria: Anthozoa: Hexacorallia): a molecular examination and description of a new species from the waters of British Columbia. Mar Biodiv 40 (4) 249–260.

[63] Ocaña O, Brito A (2004) A review of Gerardiidae (Anthozoa: Zoantharia) from the Macaronesian islands and the Mediterranean sea with the description of a new species. Rev Acad Canar Cienc XV 3–4: 159–189.

[64] Weill R (1934) Contributions à l'étude des Cnidaires et de leurs nématocysts. 1. Recherches sur les nématocystes. 2. Valeur taxonomique du cnidome.- Trav. Stn. zool. Wimereux 10–11: 1–701.

[65] Hartog JC den, Van Nierop MM (1984) A study on the gut contents of six leathery turtles *Dermochelys coriacea* (Linnaeus) (Reptilia: Testudines: Dermochelyidae) from British waters and from the Netherlands. Zool Verh Leiden 1–36.

[66] Walsh GE, Bowers RL (1971) A review of Hawaiian zoanthids with descriptions of three new species. Zool J Linn Soc-Lon 50: 161–180.

[67] Grigg RW, Bayer FM (1976) Present knowledge of the systematics and zoogeography of the order Gorgonacea in Hawaii. Pac Sci 30: 167–175.

