# Supplementary material for: Diversity of Zoanthids (Anthozoa: Hexacorallia) on Hawaiian Seamounts: Description of the Hawaiian Gold Coral and Additional Zoanthids
Source: PLoS One. 2013 Jan 9;8(1):e52607. doi: 10.1371/journal.pone.0052607 (PMC3541366; doi:10.1371/journal.pone.0052607)
Supplement: Table S1 — Sequences information. n/a = not available. FS refers to the sample number in the first author collection. Due to the short length of the sequences or availability, sequences of Microzoanthus and Isozoanthus were used only in the 16S analyses presented in the fig. S1. (DOC) [file pone.0052607.s001.doc]

Table S1: Sequences information

| Sample name | Locality | Depth (m) | Collector/donator | Museum accession # | COI | Mt16SrRNA | 18S |
| --- | --- | --- | --- | --- | --- | --- | --- |
| *Epizoanthus illoricatus* | Sulawesi, Indonesia | 22 | M. Boyer | **FS193** | **AB247349** | **AY995929** | **KC218424** |
| *Epizoanthus scotinus* | Canada | n/a | J.D. Reimer | **n/a** | **HM042364** | **KC218440** | **KC218425** |
| *Epizoanthus paguricola* | NW Mediterranean Sea | 130 | H. Zibrowius | **FS171** | **AB247347** | **AY995928** | **KC218427** |
| *Epizoanthus lindhali* | Arctic | n/a | J. Pawlowski | **FS350** | **EF672677** | **EF687816** | **KC218426** |
| *Epizoanthus ramosus* | Japan | n/a | J.D. Reimer | **n/a** | **HM040874** | **HM040878** | **KC218430** |
| *Epizoanthus* *arenaceus* | NW Mediterranean Sea | 23 | F. Sinniger | **FS101** | **AB247348** | **AY995926** | **KC218428** |
| *Epizoanthus* *couchii* | Irland | n/a | P. Chevaldonné | **FS353** | **n/a** | **AB247343** | **KC218429** |
| Parazoanthid “NC2” | New Caledonia | ~860 | B. Richer-de-Forges | **FS324** | **EU591615** | **EU591600** | **KC218413** |
| Parazoanthid “CORSARO72” | S Mediterranean Sea | 690 | H. Zibrowius | **FS368** | **EF672665** | **EF687824** | **KC218412** |
| *Antipathozoanthus macaronesicus* | Cape Verde, E Atlantic | 18 | P. Wirtz | **MHNG INVE 64735** | **AB247357** | **AY995931** | **KC218411** |
| *Mesozoanthus fossii* | Chile | 20 | G. Försterra | **MNHG INVE54343** | **EF672653** | **EF687822** | **KC218409** |
| *Parazoanthus "hertwigi"* | Ascension Island | n/a | S. Scott | **FS814** | **KC218397** | **n/a** | **KC218415** |
| *Parazoanthus* *elongatus* | Chile | 20 | V. Häussermann | **MNHG INVE54348** | **EF672661** | **EF687829** | **KC218414** |
| *Parazoanthus swiftii* | W Caribbean Sea | 31 | F. Sinniger | **FS197** | **AB247350** | **AY995936** | **KC218417** |
| *Parazoanthus axinellae* | NW Mediterranean Sea | 7 | F. Sinniger | **FS99** | **AB247355** | **AY995935** | **KC218416** |
| *Corallizoanthus tsukaharai* | S Japan | 194 | Okinawa aquarium | **MHNG-INVE 60951** | **KC218396** | **n/a** | **n/a** |
| *Corallizoanthus tsukaharai* | S Japan | 208 | Okinawa aquarium | **USNM1110399** | **n/a** | **EU035623** | **KC218410** |
| *Parazoanthus* sp. “Madagascar” | N Madagascar | 9 | F. Sinniger | **FS288** | **EF672664** | **EF687825** | **KC218421** |
| *Parazoanthus* sp. New Caledonia “Deep” | New Caledonia | 50 | F. Sinniger | **FS393** | **EU591624** | **EU591609** | **KC218420** |
| *Parazoanthus* sp. New Caledonia “Shallow” | New Caledonia | 5 | F. Sinniger | **FS383** | **EU591626** | **EU591607** | **KC218419** |
| *Parazoanthus puertoricense* | W Caribbean Sea | 17 | F. Sinniger | **FS211** | **AB247351** | **AY995933** | **KC218418** |
| *Hydrozoanthus* *tunicans* | W Caribbean Sea | 15 | F. Sinniger | **MNHG INVE 64730** | **EF672667** | **AY995941** | **KC218422** |
| *Hydrozoanthus antumbrosus* | W Caribbean Sea | 15 | F. Sinniger | **MNHG INVE 64732** | **AB247353** | **AY995940** | **KC218423** |
| *Savalia savaglia* | NW Mediterranean Sea | 41 | F. Sinniger | **FS94** | **AB247356** | **AY995925** | **HM044299** |
| *Savalia lucifica* | NE Pacific | n/a | E. Sala | **FS815** | **EF672658** | **EF687819** | **KC218398** |
| *Kulamanamana haumeaae* | Hawaii | 395 | A. Baco | **BPBM-D2250** | **KC218387** | **KC218431** | **KC218400** |
| *Kulamanamana haumeaae* | Hawaii | 427 | A. Baco | **NIWA-84101** | **KC218388** | **KC218432** | **KC218401** |
| *Kulamanamana haumeaae* | Hawaii | 406 | A. Baco | **MMC-T4** | **KC218386** | **n/a** | **KC218399** |
| *Zibrowius ammophilus* | Hawaii | 409 | A. Baco | **BPBM-D2252** | **KC218395** | **KC218439** | **KC218408** |
| *Zibrowius ammophilus* | Hawaii | 389 | A. Baco | **USNM1190192** | **KC218394** | **KC218438** | **KC218407** |
| *Hurlizoanthus parrishi* | Hawaii | 390 | A. Baco | **USNM1190194** | **KC218389** | **KC218433** | **KC218402** |
| *Kauluzoanthus kerbyi* | Hawaii | 536 | A. Baco | **BPBM-D2254** | **KC218391** | **KC218435** | **KC218404** |
| *Kauluzoanthus kerbyi* | Hawaii | 343 | A. Baco | **NMST-Co 1551** | **KC218392** | **KC218436** | **KC218405** |
| *Kauluzoanthus kerbyi* | Hawaii | 397 | A. Baco | **USNM1190196** | **KC218393** | **KC218437** | **KC218406** |
| *Bullagummizoanthus emilyacadiaarum* | Hawaii | 558 | A. Baco | **USNM1190198** | **KC218390** | **KC218434** | **KC218403** |
| *Microzoanthus kagerou* | Japan | 7 | T. Fujii | **n/a** | **HQ912800** | **HQ912820** | **n/a** |
| *Microzoanthus occultus* | Japan | 1 | T. Fujii | **MHNG-INVE-77144** | **HQ912812** | **HQ912860** | **n/a** |
| *Isozoanthus giganteus* | South Africa | 20 | E. Rodriguez | **n/a** | **n/a** | **GQ464867** | **n/a** |
| *Isozoanthus cf. giganteus* | South Africa | ~20 | E. Rodriguez | **n/a** | **n/a** | **GQ464868** | **n/a** |

n/a = not available, FS refers to the sample number in the first author collection, due to the short size of the sequences or availability, sequences of *Microzoanthus* and *Isozoanthus* were used only in the 16S analyses presented in the fig. S1.
