# Supplementary material for: Diversity of Zoanthids (Anthozoa: Hexacorallia) on Hawaiian Seamounts: Description of the Hawaiian Gold Coral and Additional Zoanthids
Source: PLoS One. 2013 Jan 9;8(1):e52607. doi: 10.1371/journal.pone.0052607 (PMC3541366; doi:10.1371/journal.pone.0052607)

Figure S1

ML phylogenetic tree reconstructed using the partial 16S sequences. The position of *Isozoanthus gigantus* near *Epizoanthus* is well supported. ML bootstrap values are indicated at the nodes. Only the groups supported by 50% or more bootstrap are represented. Microzoanthidae are used as outgroup.

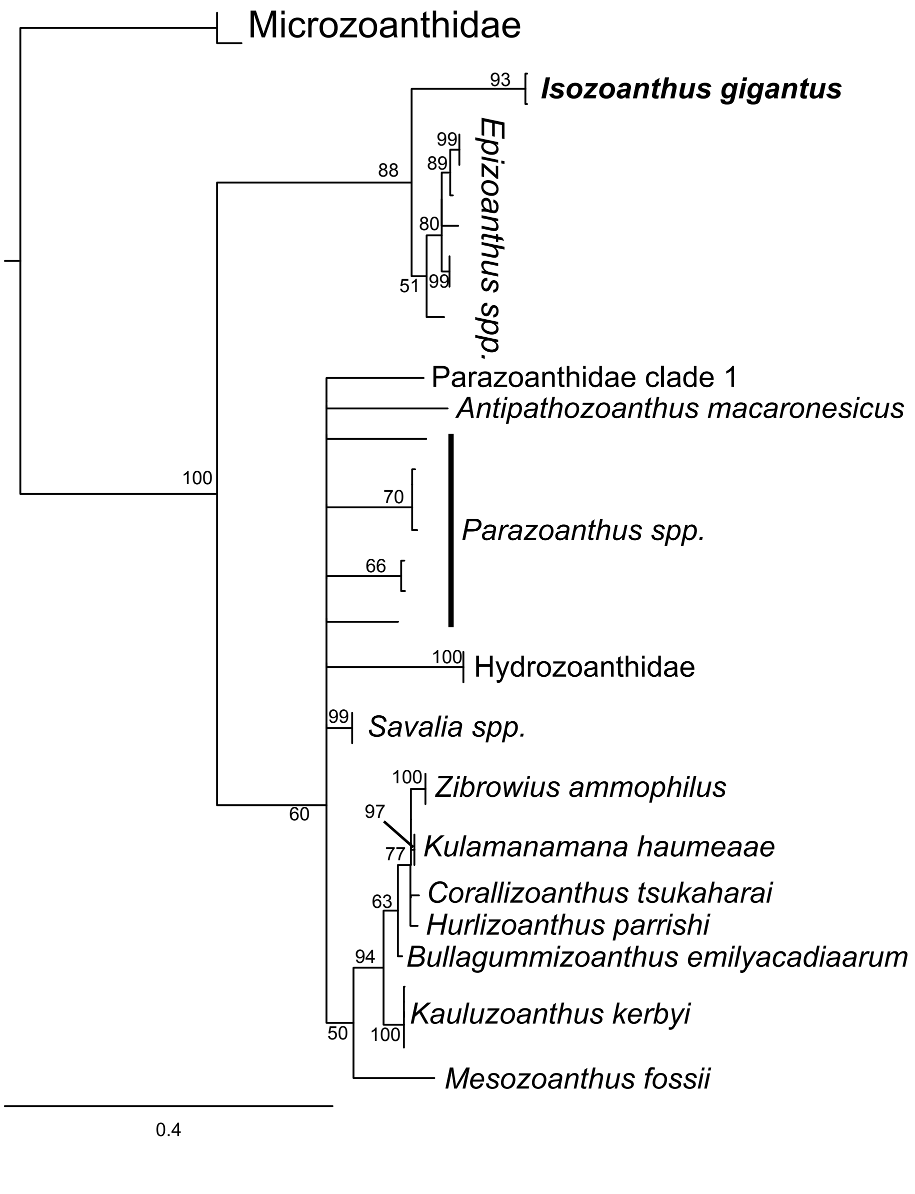

Supplement: Figure S1 — Phylogenetic tree based on 16S including Microzoanthus and Isozoanthus . ML phylogenetic tree reconstructed using the partial 16S sequences. The position of Isozoanthus gigantus near Epizoanthus is well supported. ML bootstrap values are indicated at the nodes. Only the groups supported by 50% or more bootstrap are represented. Microzoanthidae are used as outgroup. (PDF) [file pone.0052607.s002.pdf]
